# Solution of convection-diffusion model in groundwater pollution

**DOI:** 10.1038/s41598-024-52393-w

**Published:** 2024-01-24

**Authors:** Jalil Rashidinia, Arefeh Momeni, Mahboubeh Molavi-Arabshahi

**Affiliations:** Iran University of Science and Technology, School of Mathematics and Computer Science, Tehran, 16846-13114 Iran

**Keywords:** Engineering, Mathematics and computing, Physics

## Abstract

This research involves the development of the spectral collocation method based on orthogonalized Bernoulli polynomials to the solution of time-fractional convection-diffusion problems arising from groundwater pollution. The main aim is to develop the operational matrices for the fractional derivative and classical derivatives. The advantage of our approach is to orthogonalize the Bernoulli polynomials for the sake of creating sparse operational matrices in such a way that classical derivatives have one sub-diagonal non-zero entries only, and also creating an operational matrix for fractional derivative have diagonal matrix only. Due to these properties, the cost of computational our approach is very low and the convergence is fast. A discussion on the error analysis for the presented approach is given. Two test problems are considered to illustrate the effectiveness and applicability of our method. The absolute error in the computed solution compares with the existing method in the literature. The comparison shows that our method is more accurate and easily implemented.

## Introduction

Many natural phenomena such as physics, engineering, medicine, etc are modeled with fractional partial differential equations(FPDEs)^1–5^. In recent decades, many fractional devices have been developed, including thermal, mechanical, and electrical components^6^. There are many methods which are using for solving FPDEs, Including: radial basis functions(RBF)^7,8^, finite difference^9–15^, wavelets method^16–20^, spectral method^21–28^, local radial basis functions method^29,30^, finite element method^31^, Lagrange multiplier method^32^, iterative methods^33^, Crank-Nicolson method^34^. The fractional convection-diffusion equations(FCDEs) are a type of FPDEs that are widely used in various branches of science as computational mathematical models for simulations, such as: dissolved contaminant transport in groundwater, energy, and mass transformation, oil reservoir simulations, global weather, the diaspora of chemicals in reactors, etc. FCDEs of time order could be used for simulation time-related diffusion processes. Due to the importance of these equations, solving them has received much attention from researchers.

**Figure 1:**
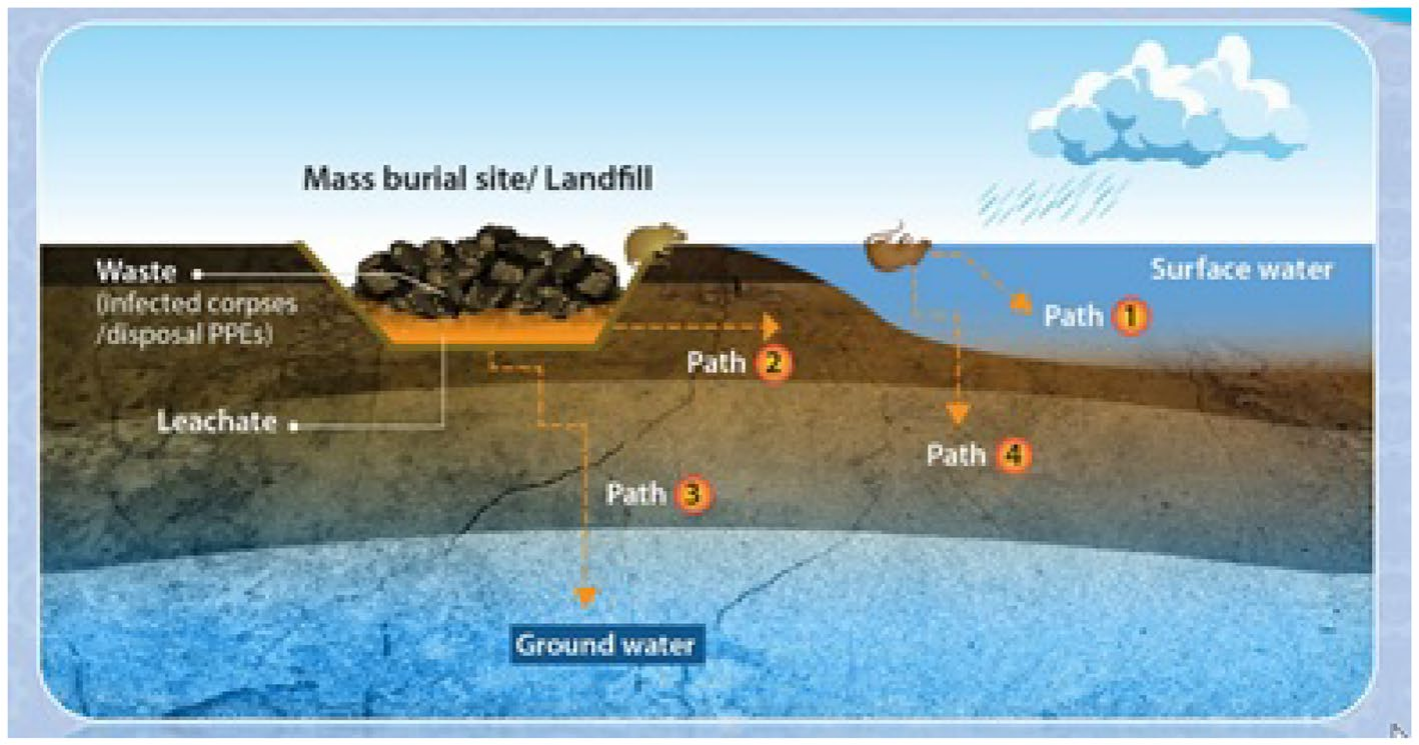
Landfill is one of the sources of groundwater pollution.

In many countries, groundwater is one of the main and most suitable sources for water supply in terms of quality and quantity. For this reason, it must be well protected and maintained. Groundwater pollution occurs as a result of human activities in several ways: Contaminated water penetrates the watershed layer in various ways from a volume of surface contaminated water (Such as leaking sewer pipes). Similarly, leakage from the rubbish heap is also a source of pollution. Contamination may also enter the soil insoluble in water (such as oil) and by gradual dissolution due to water infiltration or passage of groundwater flow causes groundwater pollution (Fig. 1). Groundwater protection is an issue with both economic and social significance^35^, so to simulate the movement of the contaminated groundwater, lots of mathematical models have been applied extensively and numerical simulation is used to solve these models. At present, many researches have been conducted on groundwater pollution problems^36–41^.

The convection-diffusion phenomenon in the research of environmental protection is often met in fluid mechanics. In the research of fluid science, the convection-diffusion equation is a consolidation of the diffusion and convection equations and describes chemical / physical phenomena where particles, energy, or other chemical / physical quantities are transferred inside a chemical / physical system due to two processes: diffusion and convection. Under some contexts, it could be also called the advection-diffusion equation, Smoluchowski equation, drift-diffusion equation^42^ or scalar transport equation^43^.

In this research, we aim to develop an efficient approach for the time fractional convection-diffusion equation (TFCDE):

1 $$\begin{aligned} D^{\gamma }_{t}u(\xi ,t)+d(\xi )\dfrac{\partial ^{2}u(\xi ,t)}{\partial \xi ^{2}}+b(\xi )\dfrac{\partial u(\xi ,t)}{\partial \xi }=r(\xi ,t),\,\,\,(\xi ,t)\in \Theta , \end{aligned}$$

subjected to the initial condition

2 $$\begin{aligned} u(\xi ,0)=g(\xi ),\,\,\,\, 0\le \xi \le 1, \end{aligned}$$

and the boundary conditions

3 $$\begin{aligned} u(0,t)=g _{0} (t),\,\,u(1,t)=g _{1} (t),\,\,\, 0< t\le 1, \end{aligned}$$

where $D^{\gamma }_{t}$ is Caputo derivative with $0<\gamma \le 1$ and $\Theta =[0,1]\times (0,1]$. sufficiently smooth functions $g, g_{0}, g_{1}$ prescribed. The TFCDE in (1) contain the particular cases:

- If $d(\xi )=-1, b(\xi )=0$, Eq. (1) is the time fractional diffusion equation(TFDE). This type of equation governs the evolution of the probability density function that describes anomalously diffusing particles.
- If $d(\xi )=-1$, Eq. (1) is the time fractional advection diffusion equation(TFADE). This equation can be solved to determine the changes in tracer concentration with space and time. Also used for water mass and marine particle transport modeling and sediment diagenesis.

Many researchers trying to solve the above-mentioned equation. Using the Chebyshev wavelet collocation method proposed in^44^. The radial basis function (RBF) method combined with a modification of the finite integration method (FIM) derived in^45^ . The sinc–Galerkin method is proposed in^46^. The Sinc–Legendre collocation method proposed in^47^. The Chebyshev collocation method was developed in^48^. Wherever the Eq. (1) be the constant coefficients, in^49^ collocation method based on RBF is developed. In the case, when $d(\xi )=-1$ and $b(\xi )=0$, authors used finite difference and finite element^50^.

In this research, we propose a new approach for the solution of TFCDEs. Our approach is to develop the operational matrices based on the orthogonalized Bernoulli polynomials. Our approach is based on the spectral collocation method based on orthogonalized Bernoulli polynomials and developing operational matrices for derivatives which is very sparse and have one sub-diagonal non-zero and also developing an operational matrix for fractional derivative which is only diagonal matrix. Due to these properties, the method is very fast, and computational time is low. Numerical experiments demonstrate the accuracy and efficiency of the proposed method.

The layout of this work is as follows:

In Section “Preliminary definitions”, some preliminary definitions are given. In Section “The proposed method”, we developed an approach for the solution of the Eq. (1)–(3). In Section “Error analysis”, we study the error analysis of the proposed method. In Section “Numerical results”, the presented method tested on two problems for verification, applicability and to show the nature of accuracy of the proposed method. We compare our results with the references^45,47,48,51–53^.

## Preliminary definitions

### Fractional derivative

By following^54^, we recall the essential concepts:

#### Definition 1

The Riemann-Liouville fractional integral operator of order $\gamma$,$(\gamma \ge 0)$ , is defined as

4 $$\begin{aligned} I^{\gamma }_{t}u(t)=\dfrac{1}{\Gamma (\gamma )}\int _{0}^{t}(t-\eta )^{\gamma -1}u(\eta )d\eta ,\,\,\,t>0, \end{aligned}$$

with $$I^{0}_{t}u(t)=u(t)$$.

#### Definition 2

The fractional derivative of *u*(*t*) in the Caputo derivative is defined as

5 $$\begin{aligned} D^{\gamma }_{t}u(t)=\dfrac{1}{\Gamma (m-\gamma )}\int _{0}^{t}(t-\eta )^{m-\gamma -1}\dfrac{\partial ^{m} u(\eta )}{\partial \eta ^{m}}d\eta , \end{aligned}$$

for $$m-1 <\gamma \le m \in \mathbb {N},\,\, t>0$$.

#### Definition 3

According to Definition 2, the time-fractional derivatives operator of order $\gamma >0$ for the function *u* is obtained by

$$\begin{aligned} D_{t}^{\gamma }u(\xi ,t)= \left\{ \begin{array}{ll} \dfrac{1}{\Gamma (m-\gamma )}\int _{0}^{t}(t-\eta )^{m-\gamma -1}\dfrac{\partial ^{m} u(\xi ,\eta )}{\partial \eta ^{m}}d\eta , &{} \,\,\,,m-1<\gamma <m,\\ \\ \dfrac{\partial ^{m} u(\xi ,t)}{\partial t^{m}}, &{} \,\,\,,\gamma =m, \end{array}\right. \end{aligned}$$

In a special case

6 $$\begin{aligned} D_{t}^{\gamma }t^{m}= \left\{ \begin{array}{ll} \dfrac{\Gamma (m+1)}{\Gamma (m+\gamma +1)}t^{m-\gamma }, &{} \,\,\,,m\ge \gamma , \\ \\ 0, &{} \,\,\,,m< \gamma , \end{array}\right. \end{aligned}$$

### The Bernoulli polynomials

#### Definition 4

The Bernoulli polynomials $(B_{k}(t))$ of *p* th degree are defined as:

7 $$\begin{aligned} \sum _{k=0}^{p} \left( {\begin{array}{c}p+1\\ k\end{array}}\right) B_{k}(t)=(p+1)t^{p},\,\,\,\,\,\, p=0,1,\ldots \end{aligned}$$

These polynomials have useful properties that we do not express here; for further information see^55,56^. Despite that, these polynomials have interested properties but are not orthogonal. To overcome such disadvantages of Bernoulli, by using the Gram - Schmidt process, we try to obtain an explicit form of orthogonal Bernoulli polynomials.

#### Definition 5

The explicit form of orthogonal Bernoulli polynomials (OBPs) of *p* th degree is as follows^57^:

8 $$\begin{aligned} \omega _{p}(t)=\sqrt{2p+1}\sum _{k=0}^{p}(-1)^{k} \left( {\begin{array}{c}p\\ k\end{array}}\right) \left( {\begin{array}{c}2p-k\\ p-k\end{array}}\right) t^{p-k},\,\,\,\,p=0,1,2,\ldots \end{aligned}$$

so that

9 $$\begin{aligned} \int _{0}^{1}\omega _{i}(t)\omega _{j}(t)dt = \delta _{ij},\,\,\,\,\,\,i,j=0,1,\ldots., \end{aligned}$$

where $\delta _{ij}$ denotes the Kronecker delta function. The form of operational matrix based on the orthogonal Bernoulli polynomial is as follows:

10 $$\begin{aligned} \Omega _{p}(t)=A\psi _{p}(t), \end{aligned}$$

where

11 $$\begin{aligned} \Omega _{p}(t)=\left[ \omega _{0}(t),\omega _{1}(t),\ldots,\omega _{p}(t)\right] ^{T},\,\,\,\psi _{p}(t)=\left[ 1,t,t^{2},\ldots,t^{p}\right] ^{T}, \end{aligned}$$

and

12 $$\begin{aligned} A= \begin{bmatrix} (-1)^{0} \left( {\begin{array}{c}0\\ 0\end{array}}\right) \left( {\begin{array}{c}0\\ 0\end{array}}\right) &{} 0 &{} \ldots &{} 0 \\ \sqrt{3}(-1)\left( {\begin{array}{c}1\\ 1\end{array}}\right) \left( {\begin{array}{c}1\\ 0\end{array}}\right) &{} \sqrt{3}(-1)^{0}\left( {\begin{array}{c}1\\ 0\end{array}}\right) \left( {\begin{array}{c}2\\ 1\end{array}}\right) &{} \ldots &{} 0\\ \vdots &{} \vdots &{} \vdots &{} \vdots \\ \sqrt{2p+1}(-1)^{p}\left( {\begin{array}{c}p\\ p\end{array}}\right) \left( {\begin{array}{c}p\\ 0\end{array}}\right) &{} \sqrt{2p+1}(-1)^{p-1}\left( {\begin{array}{c}p\\ p-1\end{array}}\right) \left( {\begin{array}{c}p+1\\ 1\end{array}}\right) &{}\ldots &{} \sqrt{2p+1}(-1)^{0}\left( {\begin{array}{c}p\\ 0\end{array}}\right) \left( {\begin{array}{c}2p\\ p\end{array}}\right) \\ \end{bmatrix}. \end{aligned}$$

Since *A* is a lower triangular matrix with nonzero diagonal elements. Therefore *A* is nonsingular. Thus, we have

13 $$\begin{aligned} \psi _{p}(t)=A^{-1}\Omega _{p}(t). \end{aligned}$$

Any function *u*(*t*) defined on (0, 1] can be expand by:

14 $$\begin{aligned} u(t)=\sum _{k=0}^{+\infty }\beta _{k}\omega _{k}(t), \end{aligned}$$

where the coefficients

15 $$\begin{aligned} \beta _{k}=\int _{0}^{1}u(t)\omega _{k}(t)dt,\,\,\,\,\,k=0,1,\ldots. \end{aligned}$$

In the application, we consider only the first $(p+1)$-terms OPBs, so that, we could write

16 $$\begin{aligned} u(t)=u_{p}(t)=\sum _{k=0}^{p}\beta _{k}\omega _{k}(t)=B^{T}\Omega _{p}(t), \end{aligned}$$

where $$B^{T}=\left[ \beta _{0},\beta _{1},\ldots,\beta _{p}\right]$$ and $$\Omega _{p}(t)=\left[ \omega _{0}(t),\omega _{1}(t),\ldots,\omega _{p}(t)\right] ^{T}$$.

In same manner, any two variables function $u(\xi ,t)$ can be expand by the OPBs series as:

17 $$\begin{aligned} u(\xi ,t)=u_{p,q}(\xi ,t)=\sum _{i=0}^{p}\sum _{j=0}^{q}h_{ij}\omega _{i}(\xi )\omega _{j}(t)=\Omega ^{T}_{p}(\xi )H \Omega _{q}(t), \end{aligned}$$

where $$H=\left[ h_{ij}\right] _{(p+1)\times (q+1)}$$ and

$$\begin{aligned} h_{ij}=\int _{0}^{1}\int _{0}^{1}\omega _{i}(\xi )u(\xi ,t)\omega _{j}(t)dt d\xi ,\,\,\,\,\, i=0,1,\ldots,p,\,\,\, j=0,1,\ldots,q, \end{aligned}$$

and

18 $$\begin{aligned} \Omega _{p}(\xi )=\left[ \omega _{0}(\xi ),\omega _{1}(\xi ),\ldots,\omega _{p}(\xi )\right] ^{T},\,\,\,\,\,\,\Omega _{q}(t)=\left[ \omega _{0}(t),\omega _{1}(t),\ldots,\omega _{q}(t)\right] ^{T}. \end{aligned}$$

According to Eq. (10), we have

19 $$\begin{aligned} \Omega _{p}(\xi )=A\psi _{p}(\xi ),\,\,\,\,\,\, \Omega _{q}(t)=A\psi _{q}(t). \end{aligned}$$

where

20 $$\begin{aligned} \psi _{p}(\xi )=\left[ 1,\xi ,\xi ^{2},\ldots,\xi ^{p}\right] ^{T},\,\,\,\,\,\,\, \psi _{q}(t)=\left[ 1,t,t^{2},\ldots,t^{q}\right] ^{T}, \end{aligned}$$

and according to Eq. (13), we get

21 $$\begin{aligned} \psi _{p}(\xi )=A^{-1}\Omega _{p}(\xi ),\,\,\,\,\,\, \psi _{q}(t)=A^{-1}\Omega _{q}(t). \end{aligned}$$

## The proposed method

We develop the spectral collocation scheme based on orthogonal Bernoulli polynomials in both the space and time direction.

- **Step 1.** In this step, using the operational matrix based on orthogonal Bernoulli polynomials approximate $D^{\gamma }_{t}u(\xi ,t)$. By using Eq. (17), we have
  22 $$\begin{aligned} D_{t}^{\gamma }u(\xi ,t)= & {} D_{t}^{\gamma }(\Omega _{p}^{T}(\xi )H \Omega _{q}(t))=\Omega _{p}^{T}(\xi )HD^{\gamma }_{t}\Omega _{q}(t), \end{aligned}$$
  according to Eqs. (6) and (19) to (21), we have
  23 $$\begin{aligned} D_{t}^{\gamma }\Omega _{q}(t)=AM_{t}A^{-1}\Omega _{q}(t). \end{aligned}$$
  where
  $$\begin{aligned} M_{t}= \begin{bmatrix} 0 &{} 0 &{} 0 &{} \ldots &{} 0 \\ 0 &{} \dfrac{1}{\Gamma (2-\gamma )}t^{-\gamma } &{} 0 &{} \ldots &{} 0 \\ 0 &{} 0 &{} \dfrac{2!}{\Gamma (3-\gamma )}t^{-\gamma } &{} \ldots &{}0\\ \vdots &{} \vdots &{}\vdots &{}\ddots &{}\vdots \\ 0 &{} 0 &{}0&{}\ldots &{} \dfrac{q!}{\Gamma (q+1-\gamma )}t^{-\gamma } \\ \end{bmatrix}_{(q+1)\times (q+1)}. \end{aligned}$$
  Now by replacing the above equation in Eq. (22), we obtain
  24 $$\begin{aligned} D_{t}^{\gamma }u(\xi ,t)=\Omega _{p}^{T}(\xi )HAM_{t}A^{-1}\Omega _{q}(t). \end{aligned}$$
- **Step 2.** In step 2, using the operational matrix based on orthogonal Bernoulli polynomials approximate $\dfrac{\partial u(\xi ,t)}{\partial t}$. By using Eqs. (17)–(19), we have
  25 $$\begin{aligned} \dfrac{\partial u(\xi ,t)}{\partial \xi }= & {} \dfrac{\partial }{\partial \xi }(\Omega ^{T}_{p}(\xi )H\Omega _{q}(t))=\dfrac{\partial }{\partial \xi }(\Omega ^{T}_{p}(\xi ))H\Omega _{q}(t)\nonumber \\= & {} \dfrac{\partial }{\partial \xi }(A\psi _{p}(\xi ))^{T}H\Omega _{q}(t)=( A\dfrac{\partial }{\partial \xi }\psi _{p}(\xi ))^{T}H\Omega _{q}(t). \end{aligned}$$
  On the other hand, by using Eq. (20), we obtain
  26 $$\begin{aligned} \dfrac{\partial }{\partial \xi }\psi _{p}(\xi )= D_{1}\psi _{p}(\xi ) \end{aligned}$$
  where
  $$\begin{aligned} D_{1}= \begin{bmatrix} 0 &{} 0 &{} \ldots &{} 0 &{}0\\ 1 &{} 0 &{} \ldots &{} 0&{}0 \\ 0 &{} 2 &{} \ldots &{}0&{}0\\ \vdots &{}\vdots &{}\ddots &{}\vdots &{}\vdots \\ 0 &{}0&{}\ldots &{}p&{} 0 \\ \end{bmatrix}_{(p+1)\times (p+1)} \end{aligned}$$
  By using Eqs. (21), (25) and (26), we have
  27 $$\begin{aligned} \dfrac{\partial u(\xi ,t)}{\partial \xi }=\Omega ^{T}_{p}(\xi )A^{-T}D^{T}_{1}A^{T}H\Omega _{q}(t). \end{aligned}$$
- **Step 3.** In this step, using the operational matrix based on orthogonal Bernoulli polynomials and $\dfrac{\partial u(\xi ,t)}{\partial \xi }$ in step 2, we approximate $\dfrac{\partial ^{2} u(\xi ,t)}{\partial \xi ^{2}}$ . So, by using Eqs. (17) and (19), we obtain
  28 $$\begin{aligned} \dfrac{\partial ^{2}u(\xi ,t)}{\partial \xi ^{2}}= & {} \dfrac{\partial ^{2}}{\partial \xi ^{2}}(\Omega ^{T}_{p}(\xi )H\Omega _{q}(t))= \dfrac{\partial ^{2}}{\partial \xi ^{2}}(\Omega ^{T}_{p}(\xi ))H\Omega _{q}(t)\nonumber \\= & {} \dfrac{\partial ^{2}}{\partial \xi ^{2} }(A\psi _{p}(\xi ))^{T}H\Omega _{q}(t)=( A\dfrac{\partial ^{2}}{\partial \xi ^{2}}\psi _{p}(\xi ))^{T}H\Omega _{q}(t). \end{aligned}$$
  by using Eq. (26), we have
  29 $$\begin{aligned} \dfrac{\partial ^{2}}{\partial \xi ^{2}}\psi _{p}(\xi )=D_{2}\psi _{p}(\xi ), \end{aligned}$$
  where
  $$\begin{aligned} D_{2}= \begin{bmatrix} 0 &{} 0 &{}&{} \ldots &{} 0&{}0 &{}0\\ 0 &{} 0 &{}&{} \ldots &{} 0 &{}0&{}0\\ 2 &{} 0 &{}&{} \ldots &{} 0&{}0&{}0 \\ 0 &{} 6 &{}&{} \ldots &{}0&{}0&{}0\\ \vdots &{}&{}\vdots &{}\ddots &{}\vdots &{}\vdots \\ 0 &{}0&{}&{}\ldots &{}p(p-1)&{}0&{} 0 \\ \end{bmatrix}_{(p+1)\times (p+1)}. \end{aligned}$$
  Now by replacing Eq. (29) in Eq. (28), we obtain
  30 $$\begin{aligned} \dfrac{\partial ^{2}u(\xi ,t)}{\partial \xi ^{2}}=\Omega ^{T}_{p}(\xi )A^{-T}D^{T}_{2}A^{T}H\Omega _{q}(t). \end{aligned}$$
- **Step 4.** In this step, by replacing Eqs. (24), (27) and (30) in Eqs. (1)–(3), we obtain following system:
  $$\begin{aligned} R_{1}(\xi ,t)= & {} \Omega ^{T}_{p}(\xi )HAM_{t}A^{-1}\Omega _{q}(t)+d(\xi )\Omega ^{T}_{p}(\xi )A^{-T}D^{T}_{2}A^{T}H\Omega _{q}(t)\\{} & {} +b(\xi )\Omega ^{T}_{p}(\xi )A^{-T}D^{T}_{1}A^{T}H\Omega _{q}(t)-r(\xi ,t)=0,\\ R_{2}(\xi )= & {} \Omega ^{T}_{p}(\xi )H\Omega _{q}(0)-g(\xi )=0,\\ R_{3}(t)= & {} \Omega ^{T}_{p}(0)H\Omega _{q}(t)-g_{0}(0)=0,\\ R_{4}(t)= & {} \Omega ^{T}_{p}(1)H\Omega _{q}(t)-g_{1}(t)=0, \end{aligned}$$
- **Step 5.**In step 5, we collocate the above system with the points $\xi _{i}= \dfrac{2i+1}{2p+2}$ and $t_{j}=\dfrac{2j+1}{2q+2}$ to obtain
  31 $$\begin{aligned} R_{1}(\xi _{i},t_{j})= & {} 0,\,\,\,\,\,\,\,\,\,\,\,i=0,\ldots,p-2,\,\,\,j=0,\ldots,q-1,\nonumber \\ R_{2}(\xi _{i})= & {} 0,\,\,\,\,\,\,\,\,\,\,\,i=0,\ldots,p,\nonumber \\ R_{3}(t_{j})= & {} 0,\,\,\,\,\,\,\,\,\,\,\,j=0,\ldots,q-1,\nonumber \\ R_{4}(t_{j})= & {} 0,\,\,\,\,\,\,\,\,\,\,\,j=0,\ldots,q-1. \end{aligned}$$
  by solving the above system of equations, we obtain the coefficients matrix *H* .
- **Step 6.** Finally, by replacing matrix *H* in Eq. (17), we approximate the solution of TFCDE.

## Error analysis

We aim to obtain an estimation of the error bound for the approximation of the function $u(\xi ,t)\in \Theta =[0,1]\times (0,1]$ with orthogonal Bernoulli polynomials.

Suppose that $f(\xi ,t)=D^{\gamma }_{t}u(\xi ,t)$ be a function on $\Theta$ and $P_{p,q}(\xi ,t)$ be the interpolating polynomials of $f(\xi ,t)$ at points $(\xi _{i},t_{j})$ , where $\xi _{i}$ , $i=0,\ldots,p$ are the roots of the $(p+1)$ -degree for orthogonal Bernoulli polynomials in [0, 1] and $t_{j}$ , $j=0,\ldots,q$ are the zeros of the $(q+1)$ -degree orthogonal Bernoulli polynomials in (0, 1] . Then we obtain

32 $$\begin{aligned} f(\xi ,t)-P_{p,q}(\xi ,t)= & {} \dfrac{\partial ^{p+1}f(\xi '_{1},t)}{\partial \xi ^{p+1}(p+1)!}\prod _{i=0}^{p}(\xi -\xi _{i})+ \dfrac{\partial ^{q+1}f(\xi ,\eta '_{1})}{\partial t^{q+1}(q+1)!}\prod _{j=0}^{q}(t-t_{j})\nonumber \\{} & {} -\dfrac{\partial ^{p+q+2}f(\xi '_{2},\eta '_{2})}{\partial \xi ^{p+1}\partial t^{q+1}(p+1)!(q+1)!}\prod _{i=0}^{p}(\xi -\xi _{i})\prod _{j=0}^{q}(t-t_{j}), \end{aligned}$$

where $\xi '_{1} ,\xi '_{2} \in [0,1]$ and $\eta '_{1} , \eta '_{2} \in (0,1]$. So that

33 $$\begin{aligned} \mid f(\xi ,t)-P_{p,q}(\xi ,t)\mid\le & {} \max \mid \dfrac{\partial ^{p+1}f(\xi ,t)}{\partial \xi ^{p+1}}\mid \dfrac{\prod _{i=0}^{p}\mid \xi -\xi _{i}\mid }{(p+1)!}\nonumber \\{} & {} +\max \mid \dfrac{\partial ^{q+1}f(\xi ,t)}{\partial t^{q+1}}\mid \dfrac{\prod _{j=0}^{q}\mid t-t_{j}\mid }{(q+1)!}\nonumber \\{} & {} + \max \mid \dfrac{\partial ^{p+q+2}f(\xi ,t)}{\partial \xi ^{p+1}\partial t^{q+1}}\mid \dfrac{\prod _{i=0}^{p}\mid \xi -\xi _{i}\mid \prod _{j=0}^{q}\mid t-t_{j}\mid }{(p+1)!(q+1)!} \end{aligned}$$

where $(\xi ,t)\in \Theta$. suppose that there are real numbers $\rho _{1}$ ,$\rho _{2}$ and $\rho _{3}$ , such that

34 $$\begin{aligned}{} & {} \max \mid \dfrac{\partial ^{p+1}f(\xi ,t)}{\partial \xi ^{p+1}}\mid \le \rho _{1}, \end{aligned}$$

35 $$\begin{aligned}{} & {} \max \mid \dfrac{\partial ^{q+1}f(\xi ,t)}{\partial t^{q+1}}\mid \le \rho _{2}, \end{aligned}$$

36 $$\begin{aligned}{} & {} \max \mid \dfrac{\partial ^{p+q+2}f(\xi ,t)}{\partial \xi ^{p+1}\partial t^{q+1}}\mid \le \rho _{3}, \end{aligned}$$

by replacing Eqs. (34) to (36) into the Eq. (33) and taking into account the maximum in Eq. (33), we obtain

37 $$\begin{aligned} \mid f(\xi ,t)-P_{p,q}(\xi ,t)\mid\le & {} \dfrac{\rho _{1}}{(p+1)!}+\dfrac{\rho _{2}}{(q+1)!}\nonumber \\{} & {} +\dfrac{\rho _{3}}{(p+1)!(q+1)!}. \end{aligned}$$

### **Theorem 1**

*Let the real-valued function* $f(\xi ,t)$ *defined on* $\Theta$ *approximated by be orthogonal Bernoulli polynomials and* $f_{p,q}(\xi ,t)=\Omega _{p}^{T}(\xi )H_{1}\Omega _{q}(t)$*. Then, there exist real numbers* $\rho _{1},\rho _{2},\rho _{3}$ *such that:*

38 $$\begin{aligned} \parallel f(\xi ,t)-f_{p,q}(\xi ,t)\parallel _{2}\le & {} \dfrac{\rho _{1}}{(p+1)!}+\dfrac{\rho _{2}}{(q+1)!}\nonumber \\{} & {} +\dfrac{\rho _{3}}{(p+1)!(q+1)!}. \end{aligned}$$

### *Proof*

By considering the definition of the best approximation and Eq. (37), we have

39 $$\begin{aligned} \parallel f(\xi ,t)-f_{p,q}(\xi ,t)\parallel _{2}^{2}= & {} \int _{0}^{1}\int _{0}^{1}\mid f(\xi ,t)-f_{p,q}(\xi ,t)\mid ^{2}d\xi dt\nonumber \\ {}\le & {} \parallel f(\xi ,t)-P_{p,q}(\xi ,t)\parallel _{2}^{2}\nonumber \\\le & {} \int _{0}^{1}\int _{0}^{1}\mid f(\xi ,t)-P_{p,q}(\xi ,t)\mid ^{2}d\xi dt\nonumber \\\le & {} \int _{0}^{1}\int _{0}^{1}[ \dfrac{\rho _{1}}{(p+1)!}+\dfrac{\rho _{2}}{(q+1)!}+\dfrac{\rho _{3}}{(p+1)!(q+1)!}]^{2}d\xi dt\nonumber \\= & {} [ \dfrac{\rho _{1}}{(p+1)!}+\dfrac{\rho _{2}}{(q+1)!}+\dfrac{\rho _{3}}{(p+1)!(q+1)!}]^{2}. \end{aligned}$$

The proof is complete. $\square$

Now by setting $f(\xi ,t)=D^{\gamma }_{t}u(\xi ,t)$ and using Theorem 1, we state and proof the following Theorem.

### **Theorem 2**

*Let* $u(\xi ,t)$ *be the exact solution of TFCDE in* (1) *real-valued function having sufficiently smooth,* $u_{p,q}(\xi ,t)$ *be the computed solution by the scheme* (17)*. Then*

40 $$\begin{aligned} \parallel u(\xi ,t)-u_{p,q}(\xi ,t)\parallel _{2}\le & {} \dfrac{1}{(m-1)!\sqrt{(2m-1)2m}} \times \big (\dfrac{\rho _{1}}{(p+1)!}+\dfrac{\rho _{2}}{(q+1)!}\nonumber \\{} & {} +\dfrac{\rho _{3}}{(p+1)!(q+1)!}\big ) \end{aligned}$$

where $$m=\max {\gamma }$$.

### *Proof*

Using Theorem 1, we have:

41 $$\begin{aligned} \parallel D^{\gamma }u(\xi ,t)-\Omega _{p}^{T}(\xi )H\Omega _{q}(t)\parallel _{2}\le \dfrac{\rho _{1}}{(p+1)!}+ & {} \dfrac{\rho _{2}}{(q+1)!}+\dfrac{\rho _{3}}{(p+1)!(q+1)!}. \end{aligned}$$

Now, by using the useful properties of the Caputo derivative and Riemann- Liouville integral defined in^58^ and Eq. (41), we obtain:

42 $$\begin{aligned} \parallel u(\xi ,t)-u_{p,q}(\xi ,t)\parallel _{2}= & {} \parallel I_{t}^{m}(D^{\gamma }_{t}u(\xi ,t)-\Omega _{p}^{T}(\xi )H_{1}\Omega _{q}(t))\parallel _{2}\nonumber \\\le & {} \parallel I_{t}^{m}\parallel _{2}\parallel D^{\gamma }_{t}u(\xi ,t)-\Omega _{p}^{T}(\xi )H_{1}\Omega _{q}(t)\parallel _{2} \end{aligned}$$

Now, we know that $I_{t}^{m}$ is the operator integral Riemann - Liouville, Then this

43 $$\begin{aligned} \parallel I_{t}^{m}\parallel _{2} =\sup _{\parallel v\parallel _{2}=1}\parallel I_{t}^{m}v\parallel _{2}, \end{aligned}$$

For proof, we need to introduce an upper bound for $\parallel I_{t}^{m}\parallel _{2}$. Using the definition of the left Riemann - Liouville integral operator and Schwarz’s inequality, we have:

44 $$\begin{aligned} \parallel I_{t}^{m}v\parallel _{2}^{2}= & {} \parallel \dfrac{1}{(m-1)!}\int _{0}^{t}(t-\tau )^{m-1}v(\tau )d\tau \parallel _{2}^{2} \nonumber \\= & {} \dfrac{1}{[(m-1)!]^{2}} \parallel \int _{0}^{t}(t-\tau )^{m-1}v(\tau )d\tau \parallel _{2}^{2}\nonumber \\= & {} \dfrac{1}{[(m-1)!]^{2}} \int _{0}^{1}\mid \int _{0}^{t}(t-\tau )^{m-1}v(\tau )d\tau \mid ^{2}dt\nonumber \\\le & {} \dfrac{1}{[(m-1)!]^{2}} \int _{0}^{1}(\int _{0}^{t}(t-\tau )^{2m-2}d\tau )(\int _{0}^{1}\mid v(\tau )\mid ^{2}d\tau )dt\nonumber \\= & {} \dfrac{1}{[(m-1)!]^{2}2m(2m-1)}. \end{aligned}$$

Therefore, we have:

45 $$\begin{aligned} \parallel I_{t}^{m} \parallel _{2}\le \dfrac{1}{(m-1)!\sqrt{2m(2m-1)}}. \end{aligned}$$

Finally, by using (42) and (45), we obtain:

46 $$\begin{aligned} \parallel u(\xi ,t)-u_{p,q}(\xi ,t)\parallel _{2}\le & {} \dfrac{1}{(m-1)!\sqrt{(2m-1)2m}} \times (\dfrac{\rho _{1}}{(p+1)!}+\dfrac{\rho _{2}}{(q+1)!}\nonumber \\{} & {} +\dfrac{\rho _{3}}{(p+1)!(q+1)!}) \end{aligned}$$

Now completed the proof. $\square$

## Numerical results

Here we consider two problems to demonstrate the reliability and efficiency of our method. The absolute errors of the solution for different nodes $(\xi _{i},t_{j})\in \Theta$ defined as this:

$$\begin{aligned} e(\xi _{i},t_{j})=\mid u(\xi _{i},t_{j})-u_{p,q}(\xi _{i},t_{j})\mid , \end{aligned}$$

also, the maximum absolute errors(MAEs) are

47 $$\begin{aligned} e_{max}(\xi _{i},t_{j})=\max (\mid u(\xi _{i},t_{j})-u_{p,q}(\xi _{i},t_{j})\mid ),\,\,\, i=0,\ldots,p,\,\,\, j=0,\ldots,q. \end{aligned}$$

The presented method applied to these problems for various *p*,  *q* and $\gamma$. All the programming in Matlab 2016 software is run by a computer with core i5.

- **Problem 1.**

Consider the following TFCDE^48,51^:

48 $$\begin{aligned} D^{\gamma }_{t}u(\xi ,t)+\xi \dfrac{\partial u(\xi ,t)}{\partial \xi }+\dfrac{\partial ^{2}u(\xi ,t)}{\partial \xi ^{2}}=2t^{\gamma }+2\xi ^{2}+2,\,\,\,0<\xi <1,\,\,t\in (0,1], \end{aligned}$$

subjected to the initial and boundary Conds.

$$\begin{aligned} u(\xi ,0)= & {} \xi ^{2},\\ u(0,t)= & {} 2\dfrac{\Gamma (\gamma +1)}{\Gamma (2\gamma +1)}t^{2\gamma },\,\,u(1,t)= 1+ 2\dfrac{\Gamma (\gamma +1)}{\Gamma (2\gamma +1)}t^{2\gamma }, \end{aligned}$$

where $\,\,0<\gamma \le 1$. Exact solution is $u(\xi ,t)=\xi ^{2}+\dfrac{\Gamma (\gamma +1)}{\Gamma (2\gamma +1)}t^{2\gamma }$.

**Figure 2:**
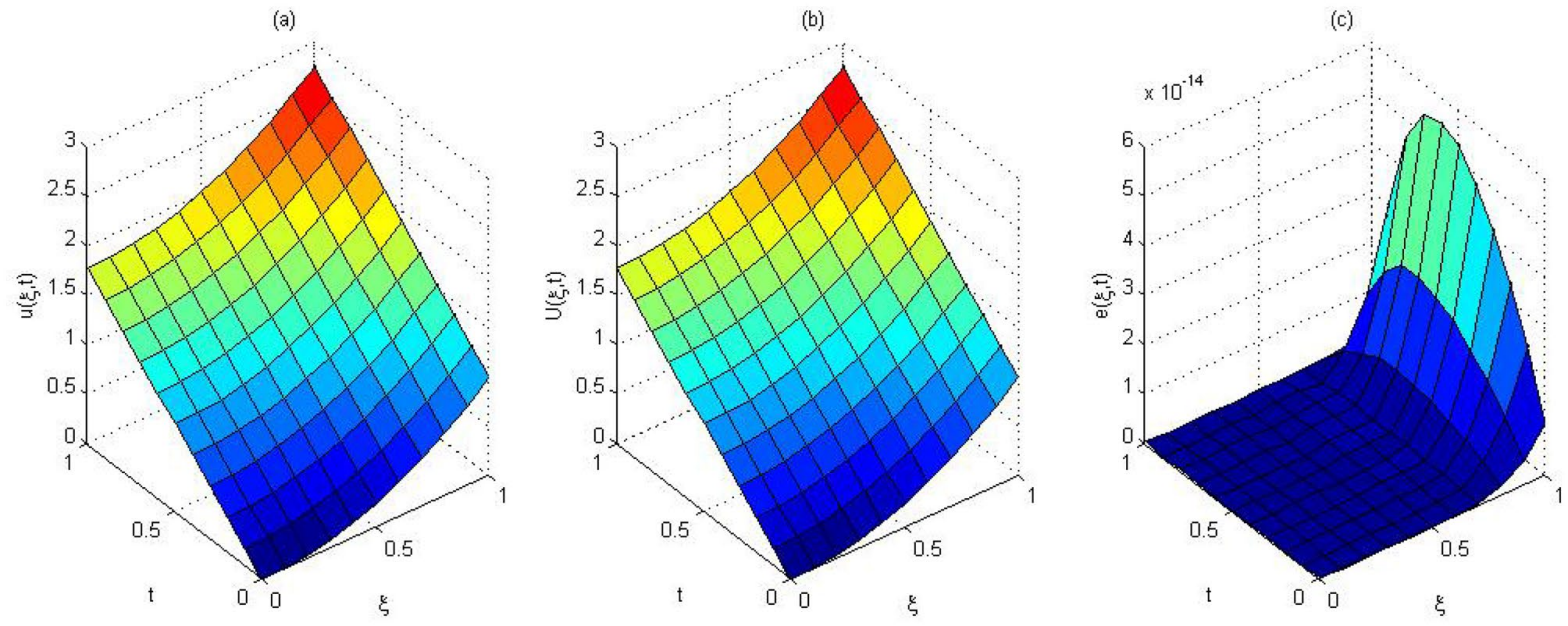
The computed solution (**a**), the exact solution (**b**), the absolute error (**c**), with $p=q=4$ and $\gamma =0.5$ for Problem 1.

**Figure 3:**
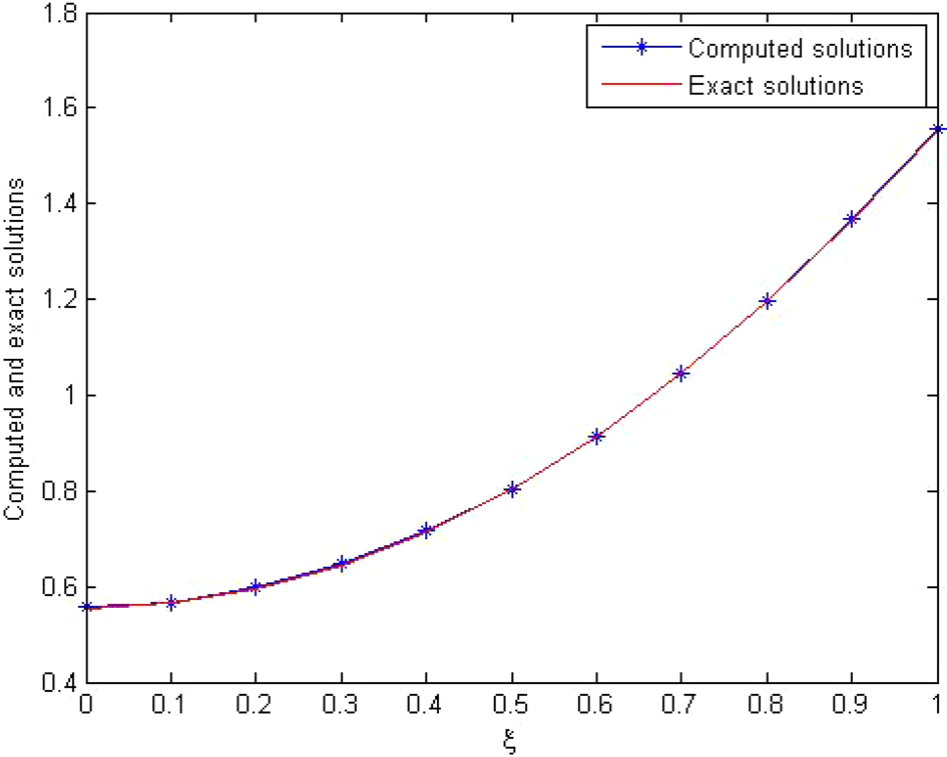
The comparison with the computed and exact solutions at $t=0.5$ with $\gamma =0.7$and $p=q=3$ for Problem 1.

**Figure 4:**
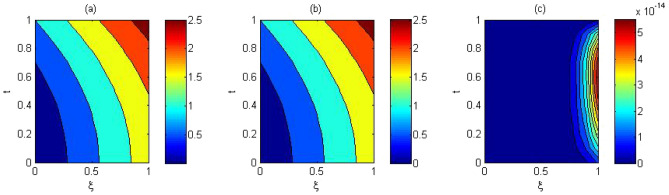
The contour plots of (**a**) computed solution. (**b**) exact solution, (**c**) the absolute error $p=q=4$ and $\gamma =0.5$ for Problem 1.

The computed solution and absolute errors in the solution for $p=q=4$ and $\gamma =0.5$ were obtained. We plot the graph of the computed solution, the exact solution, and absolute errors in solution in Fig. 2a–c respectively. In Fig. 3, we plot the computed solution and the exact solution for $p=q=3$ with $\gamma =0.7$ at $t=0.5$. We show that the computed solution coincides with the exact solution. In Fig. 4a–c the contour plots of the computed solution, the exact solution and absolute errors are represented for $p=q=4$ with $\gamma =0.5$. Moreover in Table 1, we compared the MAE of the proposed method with the methods in^45,47,48,51,52^. Table 1, shows that our method is more efficient and accurate than the compared methods. Our method for $p=q=2$ yields the solution of a system of $3\times 3$ but in^51^ for $m=5, J=3$ needs to solve a system of $110 \times 110$. The reference^52^ needs to solve the system of $32 \times 32$. The reference^47^ needs to solve the system of $31\times 8$. The reference^48^ needs to solve the system of $6\times 6$. Finally, the method in^45^ needs to use 10 grid points in time and space but our method uses 3 grid points in time and space. The absolute errors in the solution for this problem for $p=q=3$ and $p=q=4$ with $\gamma =0.5$ for $t=0.1$ and the run time of our method is tabulated to show the effectiveness of our method in Table 2.

**Table 1:** Absolute compared errors with $\gamma =0.5,t=0.5$ for Problem 1.

$\xi$	^51^	^45^	^52^	^47^	^48^	Our method
	$m=5,J=3$	$N_x=10, N_t=10$	$m=32$	$m=15, n=7$	$m=5$	$p=q=2$
0.1	6.481e−04	5.5127e−05	6.093e−03	7.964e−06	6.994e−05	1.4340e−10
0.2	4.109e−04	6.3034e−05	4.843e−03	1.721e−04	3.912e−06	1.8880e−10
0.3	5.493e−04	2.5286e−05	2.750e−02	2.472e−04	6.162e−06	2.1065e−10
0.4	5.198e−04	9.7841e−06	1.937e−02	2.912e−04	5.953e−06	2.0896e−10
0.5	4.912e−04	2.3230e−06	1.000e−06	3.004e−04	2.103e−06	1.8372e−10
0.6	5.063e−04	6.5798e−06	4.359e−02	2.760e−04	7.639e−06	1.3493e−10
0.7	5.045e−04	3.6040e−06	1.734e−02	2.213e−04	1.967e−06	6.2598e−11
0.8	5.040e−04	1.8435e−06	7.750e−02	1.440e−04	8.103e−06	3.3282e−11
0.9	5.037e−04	6.3126e−05	4.443e−02	5.026e−05	6.019e−06	1.5271e−10

**Table 2:** Absolute errors for values of $p=q=2$, $p=q=3$ and $p=q=4$, $\gamma =0.5,$ $t=0.1$ for Problem 1.

$\xi$	$p=q=2$	$p=q=3$	$p=q=4$
0.1	1.0481e−10	2.4486e−11	2.7755e−17
0.2	2.7048e−10	2.0630e−13	1.3877e−16
0.3	4.0141e−10	8.7591e−11	5.5511e−17
0.4	4.9762e−10	2.0219e−10	1.6653e−16
0.5	5.5910e−10	3.0690e−10	1.1102e−16
0.6	5.8584e−10	3.6500e−10	0
0.7	5.7786e−10	3.3978e−10	0
0.8	5.3514e−10	1.9453e−10	1.1102e−16
0.9	4.5770e−10	1.0744e−10	0
Time(s)	4.1074	16.2225	72.5018

- **Problem 2.** Consider the following TFCDE^53^:

49 $$\begin{aligned} D^{\gamma }_{\eta }u(\xi ,t)+\xi \dfrac{\partial u(\xi ,t)}{\partial \xi }-\dfrac{\partial ^{2}u(\xi ,t)}{\partial \xi ^{2}}=r(\xi ,t),\,\,\,0\le \xi \le 1,\,0<t\le 1, \end{aligned}$$

subjected to the initial condition

$$\begin{aligned} u(\xi ,0)=\xi -\xi ^{3}, \end{aligned}$$

and $u(\xi ,t)$ vanish is on bounders i.e.

$$\begin{aligned} u(0,t)=u(1,t)= 0, \end{aligned}$$

and

$$\begin{aligned} r(\xi ,t)=\dfrac{\Gamma (1+2\gamma )}{\Gamma (1+\gamma )}t^{\gamma }(\xi -\xi ^{3})+(1+t^{2\gamma })(7\xi -3\xi ^{3}), \end{aligned}$$

exact solution is $u(\xi ,t)=(1+t^{2\gamma })(\xi -\xi ^{3})$.

The absolute errors are computed. The comparison of absolute errors of the proposed method with the method in^53^ tabulated in the Table 3, shows that our method is more efficient and accurate. Actually our method for $p=q=3$ yields the solution of system of $4\times 4$ but in^53^ for $k=4, M=1$ needs to solve a system of $48 \times 21$. In Table 4, we report the absolute errors at $t=0.1$ for various $\gamma$ , $p=q=4$, and the run time of our method is tabulated to show the effectiveness of our method.

**Figure 5:**
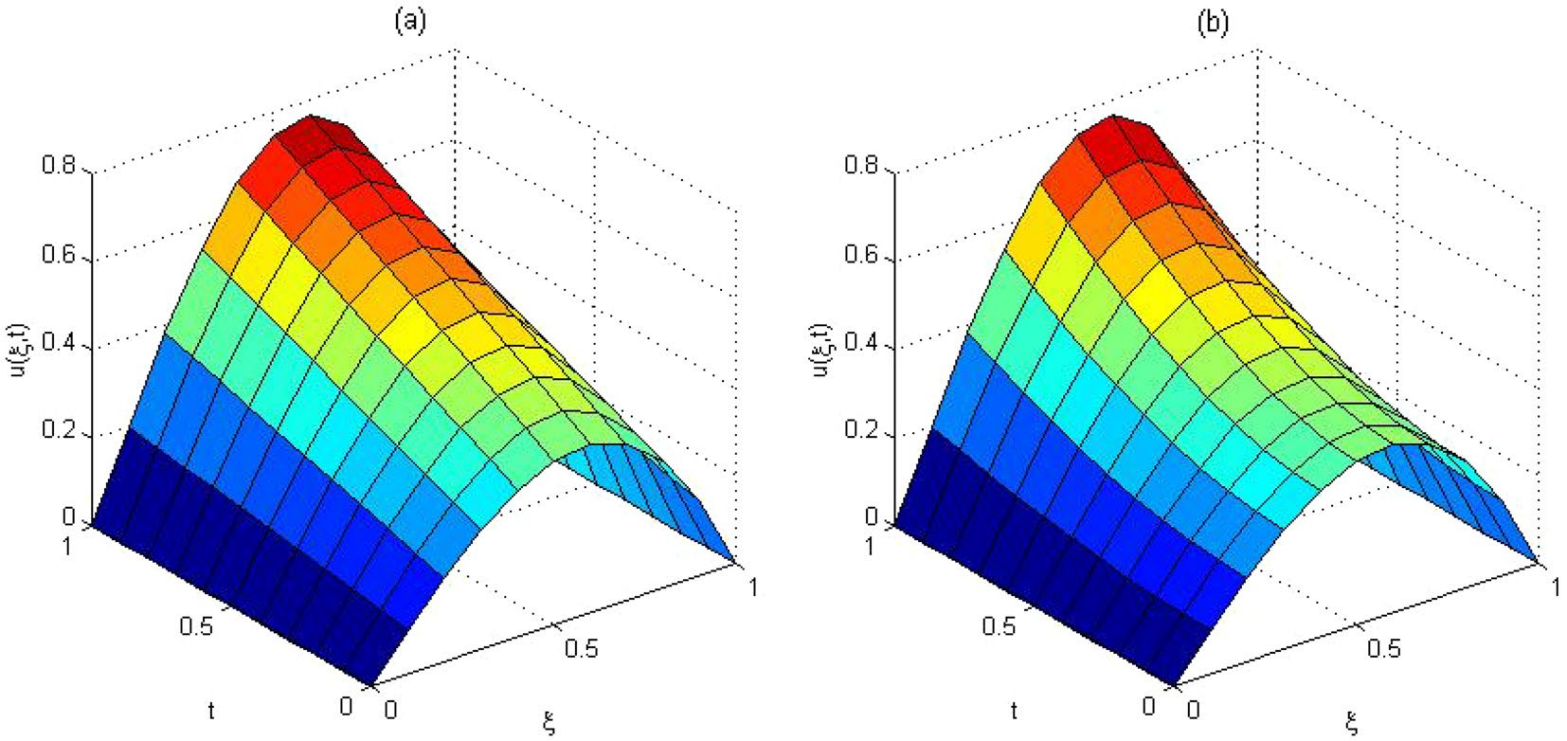
(**a**) The computed solution with $p=q=3$ and $\gamma = 0.5$ . (**b**) The computed solution with $p=q=3$ and $\gamma =0.9$ for Problem 2.

**Figure 6:**
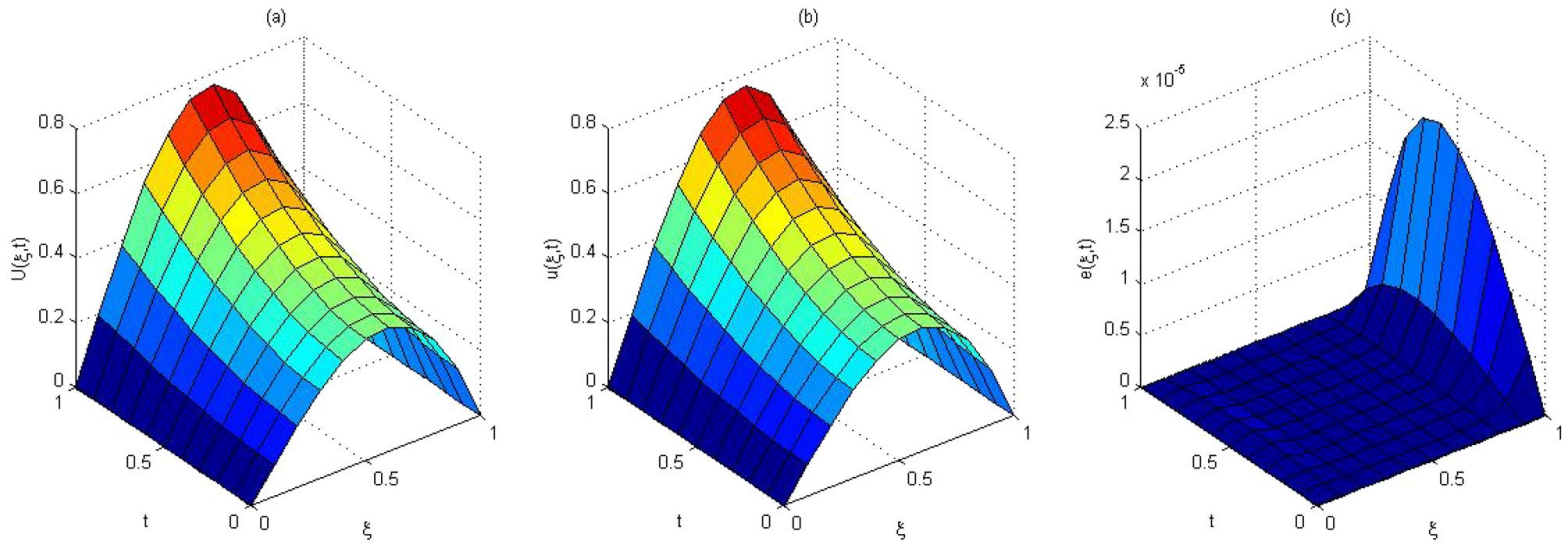
(**a**) The exact solution. (**b**) The computed solution. (**c**) the absolute error $p=q=5$ and $\gamma =0.9$ for Problem 2.

**Figure 7:**
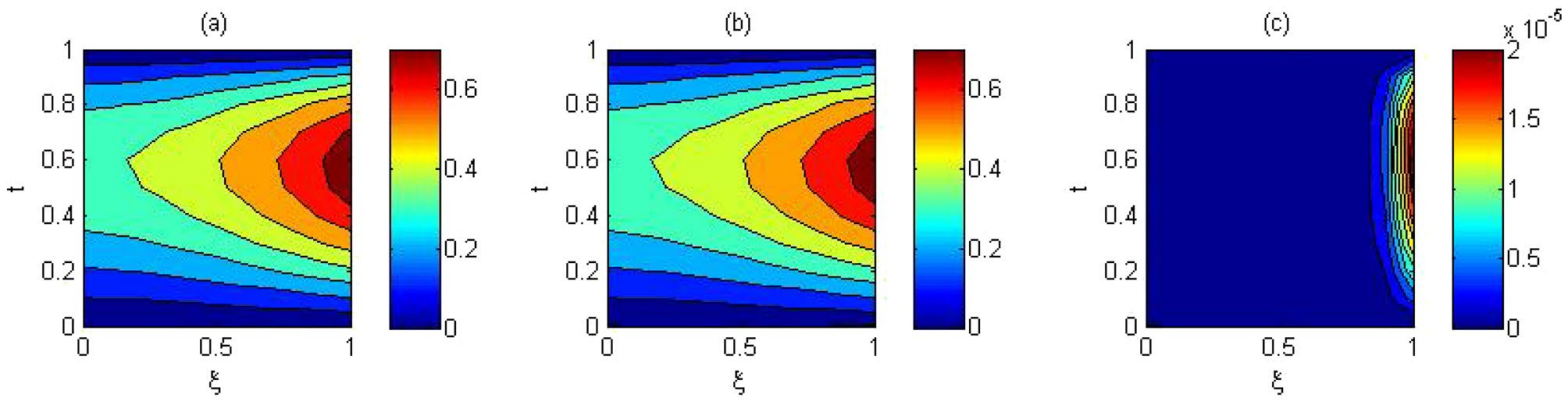
Contour plots of (**a**) computed solution. (**b**) Exact solution, (**c**) the absolute error $p=q=5$ and $\gamma =0.9$ for Problem 2.

Figure 5a,b represent the computed and the exact solutions for $p=q=5$ with $\gamma =0.5$ and $\gamma =0.9$ respectively. We plot the graph of the exact solution, the computed solution, and absolute errors in solution for $p=q=5$ and $\gamma =0.9$. in Fig. 6a–c respectively. In Fig. 7a–c the contour plots of the computed solution, the exact solution and absolute errors are represented for $p=q=5$ with $\gamma =0.9$, respectively.

**Table 3:** Absolute compared errors with $\gamma =0.7,t=0.5$ for Problem 2.

$\xi$	Our method	^53^	Our method	^53^
	$p=q=3$	$k=4,M=1$	$p=q=4$	$k=5,M=2$
0.1	1.5258 e−04	3.3203e−03	2.1877e−05	1.6340e−03
0.2	2.9665 e−04	6.4390e−03	4.2260e−05	3.1686e−03
0.3	4.2269 e−04	9.1546e−03	5.9644e−05	4.5045e−03
0.4	5.2117 e−04	1.1266e−02	7.2663e−05	5.5425e−03
0.5	5.8257 e−04	1.2571e−02	8.0089e−05	6.1836e−03
0.6	5.9737 e−04	1.2870e−02	8.0830e−05	6.3291e−03
0.7	5.5603 e−04	1.1961e−02	7.3936e−05	5.8808e−03
0.8	4.4904 e−04	9.6461e−03	5.8594e−05	4.7409e−03
0.9	2.6687 e−04	5.7250e−03	3.4127e−05	2.8126e−03

**Table 4:** Absolute errors with $p=q=4,$ and various $\gamma$ at $t=0.1$ for Problem 2.

$\xi$	$\gamma =0.3$	$\gamma =0.7$	$\gamma =0.9$
0.1	2.5024e−04	8.0124e−05	3.9414e−05
0.2	4.8248e−04	1.5450 e−04	7.5995e−05
0.3	6.7876e−04	2.1739 e−04	1.0691 e−04
0.4	8.2331e−04	2.6376 e−04	1.2968 e−04
0.5	9.0260e−04	2.8926 e−04	1.4218 e−04
0.6	9.0526e−04	2.9022 e−04	1.4261 e−04
0.7	8.2216e−04	2.6370 e−04	1.2953 e−04
0.8	6.4637e−04	2.0742 e−04	1.0184 e−04
0.9	3.7316e−04	1.1981 e−04	5.8802e−05
Time(s)	38.7477	37.1628	35.3639

## Conclusion

An efficient, accurate spectral collocation method based on orthogonalized Bernoulli polynomials has been developed for time fractional convection-diffusion problems. Our approach contains operational matrices for approximate derivatives as well as fractional derivative. Operational matrices for derivatives are sparse having one sub-diagonal non-zero entries only, and for the fractional derivatives operational matrix is diagonal only. Due to these properties and using spectral methods convergence our presented method is spectral and fast with low computation cost. The comparing numerical results justifies the effectiveness and accuracy of our proposed scheme.

## Data Availability

Te datasets used and/or analysed during the current study available from the corresponding author on reasonable request.
